# Longitudinal Evaluation of Working Memory in Duchenne Muscular Dystrophy

**DOI:** 10.3390/jcm9092940

**Published:** 2020-09-11

**Authors:** Mathula Thangarajh, Gary L. Elfring, Panayiota Trifillis

**Affiliations:** 1 Department of Neurology, Virginia Commonwealth University, 1101 East Marshall Street, P.O. Box 980599, Richmond, VA 23298, USA; 2 PTC Therapeutics Inc., South Plainfield, NJ 07080, USA; gelfring@ptcbio.com (G.L.E.); ptrifillis@ptcbio.com (P.T.)

**Keywords:** cognition, Duchenne muscular dystrophy, digit span, working memory, clinical trial

## Abstract

**Objective:** The developmental maturation of forward and backward digit spans—indices of working memory—in boys with nonsense (nm) Duchenne muscular dystrophy (DMD) (nmDMD) was assessed using prospective, longitudinal data. Methods: Fifty-five boys of the 57 subjects with genetically confirmed nmDMD—who were from the placebo arm of a 48-week-long phase 2b clinical trial—were evaluated. Forward and backward digit spans were obtained every 12 weeks for a total of five assessments in all study subjects. Changes in forward and backward digit spans were evaluated based on age, corticosteroid treatment, and DMD mutation location. **Results:** Boys with nmDMD had lower mean scores on normalized forward digit span. Normalized forward digit spans were comparable between subjects stratified by age and between corticosteroid-naïve and corticosteroid-treated subjects. When stratified by DMD mutation location, normalized forward digit spans were lower in nmDMD subjects with mutations downstream of DMD exon 30, exon 45, and exon 63, both at baseline evaluation and at follow-up evaluation at 48 weeks. On average, normalized backward digit span scores were stable over 48 weeks in these subjects. Developmental growth modeling showed that subjects with nmDMD mutations upstream of DMD exon 30, upstream of DMD exon 45, and upstream of DMD exon 63 appeared to make better gains in working memory than subjects with mutations downstream of DMD exon 30, downstream of DMD exon 45, and downstream of DMD exon 63. **Conclusion:** Performance in working memory shows deficits in nmDMD and differed based on nmDMD location. Maturation in cognition was seen over a 48-week period. The developmental trajectory of working memory in this cohort was influenced by *DMD* mutation location.

## 1. Introduction

Duchenne muscular dystrophy (DMD) is a multisystem genetic disorder affecting the brain and skeletal and cardiac muscles [1,2]. Clinically, DMD is characterized by progressive skeletal muscle weakness, which results in boys becoming wheelchair bound in their early to mid-teenage years; cardiopulmonary compromise results in death in the third-to-fourth decade of life. DMD is caused by mutations in the dystrophin gene located on the X-chromosome which affect the expression of dystrophin—a large cytoskeletal protein with myriad functional properties—in various tissues [3,4]. Through tissue- and cell-specific promoters, full-length dystrophin (dp427) and shorter dystrophin proteins (dp260, dp140, and dp71) are differentially expressed [5,6]. The unique first exons for the shorter dystrophin proteins are exon 30 for dp260, exon 45 for dp140, and exon 63 for dp71. The full-length dystrophin and shorter dystrophin proteins are expressed in the brain by neurons and astroglial cells [7,8]. In cortical neurons, dystrophin associates with postsynaptic density 95, a large scaffold protein that anchors receptors, ion channels, and other signaling molecules, to influence synaptogenesis [9]. A signaling role for dystrophin has been proposed in glial lineage cells [10,11,12]. Thus, dystrophin in the brain may influence synaptogenesis, synaptic efficiency, and synaptic tuning directly through neurons and indirectly through astroglial cells. In skeletal and cardiac muscles, dystrophin with the dystrophin–glycoprotein complex connects the intracellular actin cytoskeleton to the extracellular matrix and contributes to muscle membrane integrity [13,14,15].

Cognitive abnormalities in DMD begin early [16,17], persists [18], and are comorbid with other neurodevelopmental conditions [19,20,21]. Cotton et al. performed a meta-analysis of 1224 individuals with muscular dystrophy from 32 research studies and showed that age-related intellectual gains were most apparent in verbal intelligence compared to performance intelligence [22]. Some of the research studies included in the meta-analysis were conducted as early as the 1960s, thus limiting the ascertainment of a genetic diagnosis of DMD, as molecular testing of the dystrophin gene (*DMD*) did not become available until the late 1980s [3]. To date, published studies on cognition during childhood in DMD have been mostly cross-sectional [23,24,25,26,27,28,29,30,31]. There is a single report of 41 boys with DMD followed longitudinally over a mean of 1.5 years [18]; this study utilized two different cognitive batteries to assess neurodevelopmental outcomes at the two time points (ages 4 and 6 years). Further, previous studies have also used intelligence quotient (IQ) as an indicator of cognitive ability. IQ is not a sensitive indicator of cognition in DMD because even boys with a normal IQ have disproportionate deficit in working memory [32]. Working memory impairment in DMD is well-documented [33,34]. While previous literature suggests that cognition (as measured by IQ) may show age-related improvement, more contemporaneous scientific literature suggests that individuals with DMD may have impairments in working memory skills despite normal IQ [32]. There exists a knowledge gap in our understanding of developmental gains of core cognitive skills such as working memory in DMD. Addressing this knowledge gap will allow for more *completeness* of cognitive surveillance and rehabilitation in individuals with DMD. Identifying whether the cognitive trajectory of individuals with DMD matches that of their peers is the first critical step to plan for early and effective personalized interventions to mitigate the negative consequences of cognitive impairment on educational and vocational outcomes.

We had previously reported that subjects with nonsense *DMD* mutations (nmDMD) downstream of *DMD* exon 45 perform poorly in forward and backward digit spans, indices of working memory [34]. Non-pharmacological cognitive rehabilitation strategies targeted towards the improvement of working memory have been employed in several chronic childhood disorders [35,36,37]. We sought to prospectively evaluate the developmental trajectory of working memory in DMD. Establishing the developmental trajectory of working memory is the first critical step towards the empirical evaluation of cognitive training in DMD, as this understanding allows us to determine that the gains in working memory is due to the intervention and not simply reflective of age-related maturation. We prospectively measured forward and backward digit spans in a cohort of genetically homogenous, well-characterized cohort of 55 subjects with nmDMD enrolled in a 48-week-long phase 2b clinical trial. Our data provide scientific evidence that subjects with nmDMD show developmental gains in working memory and that the developmental trajectory of working memory is influenced by *DMD* mutation location.

## 2. Experimental Section

### 2.1. Methods

#### 2.1.1. Standard Protocol Approvals, Registrations, and Patient Consents

The parent study was approved by the local institutional review board/ethics committee at all participating sites. All participants provided signed written consent. The study conformed to the Declaration of Helsinki (2000) and the Principles of Good Clinical Practice according to the International Conference on Harmonization. The study is registered under ClinicalTrials.Gov (NCT02090959).

#### 2.1.2. Study Design and Subjects

The parent study was an international, multicenter, prospective, phase 2b clinical trial which was conducted between 2004 and 2010. Supplementary Tables S1 and S2 list the Principal Investigators and Clinical Evaluators who were involved in the clinical trial. The study design and methodology has been previously published [38]; the primary outcome of the trial was the change in 6-min walk distance in nmDMD boys treated with placebo versus ataluren, a premature termination codon readthrough molecule. Ambulatory boys inclusive of ages 5–20 years with nmDMD had digit span assessments every 12 weeks for a total of 48 weeks. In this current report, we present data from 55 of the 57 subjects with nmDMD who were in the placebo arm of the phase 2b clinical trial from whom data was available. This secondary data analysis was performed only from subjects in the placebo arm to avoid possible confounding treatment effects of ataluren.

#### 2.1.3. Study Measures

Forward and backward digit spans were evaluated as previously described [34]. Briefly, subjects hear a sequence of numerical digits and are to recall them in the same presented order (forward span) and in the reverse order (backward span). Raw scores were converted to an age-normed score using normative values [39]. Scaled scores have a population mean of 10 and a standard deviation of 3. Scaled scores for digit span are available in three-month increments beginning at age 6 years (i.e., 6 to 6.3 months, 6.4 months to 6.7 months, etc.). In the parent phase 2b clinical trial, subjects were stratified into two groups based on age into subjects less than 9 years of age and into subjects equal or older than 9 years of age. The same age categorization was maintained for the present data analysis.

#### 2.1.4. Study Participant Grouping Based on Location of nmDMD Mutation

All study participants had confirmatory gene sequencing for nmDMD. Based on their individual nmDMD mutation location, subjects were grouped into those with nm upstream or downstream of *DMD* exon 30, upstream or downstream of *DMD* exon 45, and upstream or downstream of *DMD* exon 63, as previously described [34]. Mutations upstream of any stated *DMD* exon are noted as ≤ and downstream of any stated *DMD* exon are noted as >.

### 2.2. Statistical Analyses

A student t-test was used to compare mean normalized scores between nmDMD locations. The change in mean normalized score at baseline and week 48 was performed using mixed model repeated analyses. The correlation between digit span and *DMD* genotype was analyzed using linear regression. To model developmental growth, within-subject change in normalized forward digit span over time was performed. All analyses were performed with SAS software version 9.4 (SAS Institute, Cary, NC, USA). Tests were two-sided, and the level of statistical significance was set at 0.05.

## 3. Results

### 3.1. Subject Demographics

The mean age at study enrollment for the 55 subjects (on whom data was available at baseline) was 8 years (range, 5 to 16 years). At study enrollment, 31 subjects were <9 years of age and 24 subjects were ≥9 years. The number of subjects who were corticosteroid-naïve and corticosteroid-treated were 17 and 38, respectively. The normalized forward and backward digit span scores at each of the five assessments over the course of the study are summarized in Table 1 and Table 2, respectively.

### 3.2. Developmental Change in Working Memory as a Function of Age, Oral Corticosteroid Treatment, and nmDMD Mutation Location

**Figure 1:**
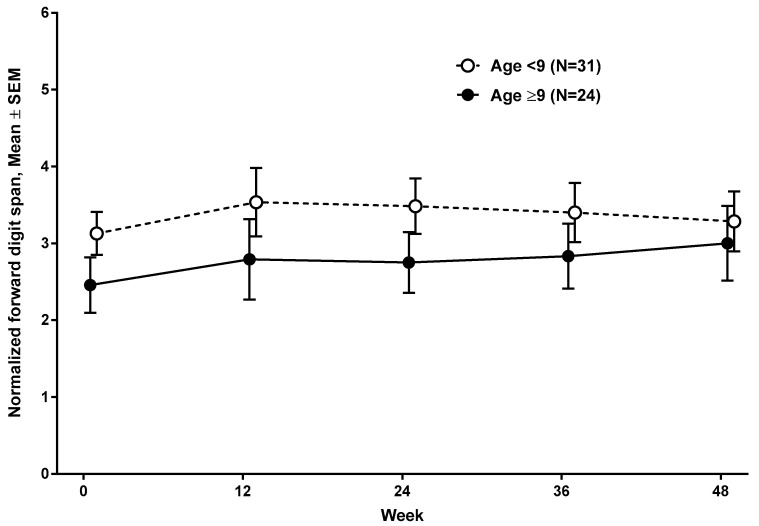
Developmental maturation of forward digit span over 48 weeks.

**Figure 2:**
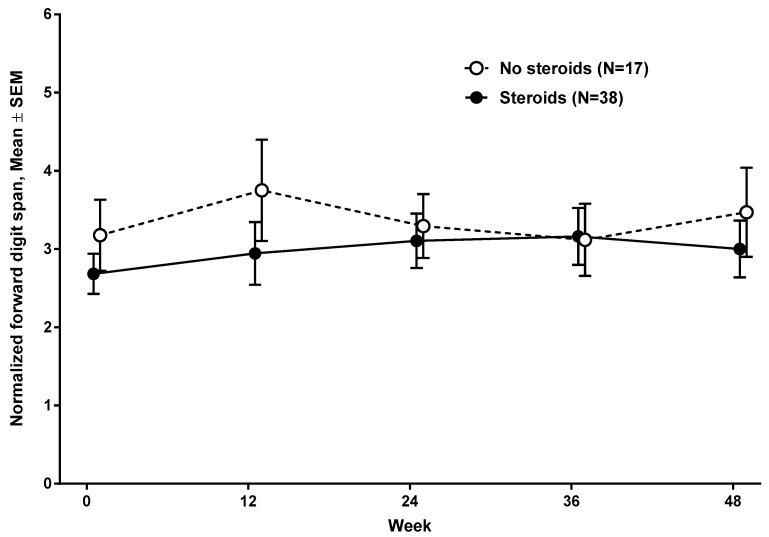
Forward digit span based on oral corticosteroid treatment.

**Figure 3:**
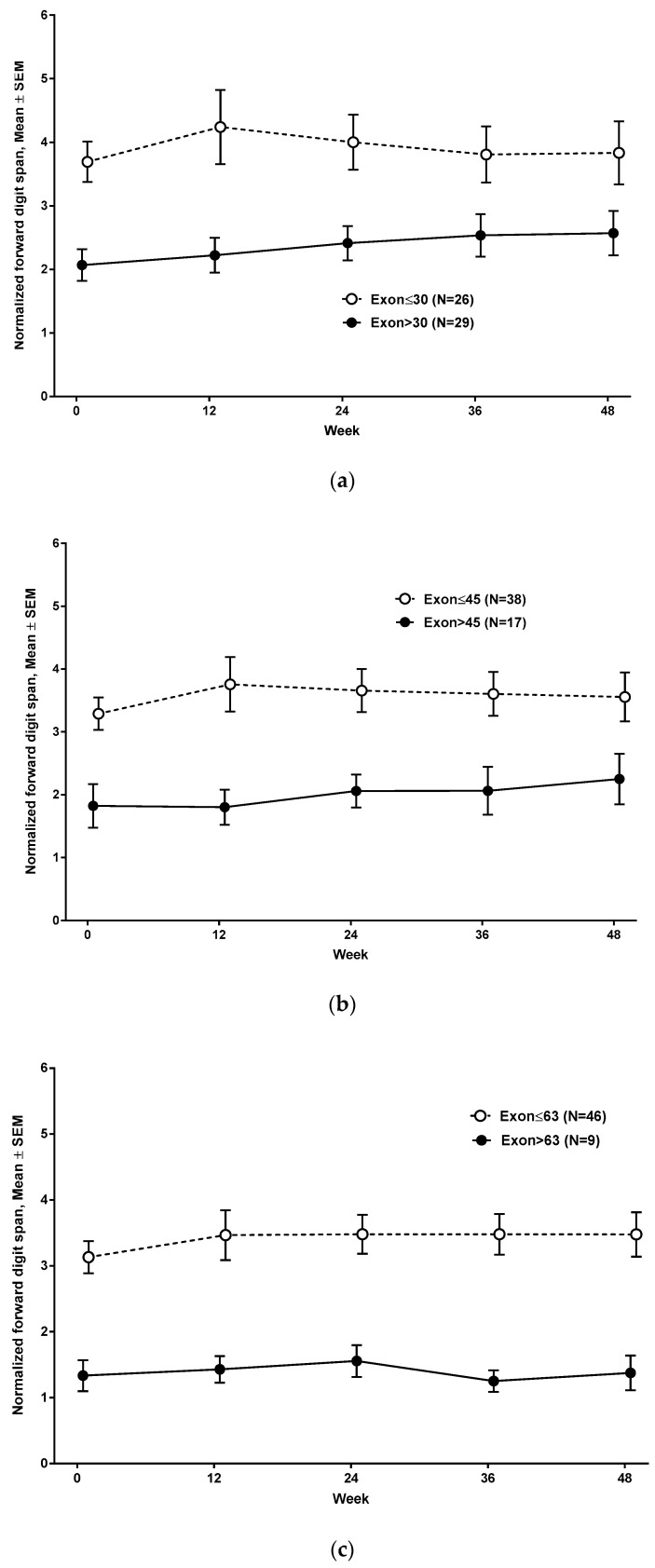
(**a**–**c**) Forward digit span based on nonsense Duchenne muscular dystrophy (nmDMD) mutation location.

At baseline evaluation, the mean normalized forward digit span was 3.13 (SD 1.57, range, 1 to 7) in subjects <9 years and 2.46 (SD 1.77, range, 1 to 6) in subjects ≥9 years (Figure 1) (n = 55). The mean normalized backward digit span was 1.30 (SD 0.7, range, 1 to 4) in subjects <9 years and 1 (SD 0, range, 1) in subjects ≥9 years. Normalized forward digit spans at baseline and at 48 weeks were comparable between subjects who were on corticosteroid treatment (n = 38), and subjects who were not on corticosteroid (n = 17) (Figure 2). Subjects with nmDMD mutations downstream of *DMD* exon 30 (n = 29), downstream of *DMD* exon 45 (n = 17), and downstream of *DMD* exon 63 (n = 9) scored lower on forward digit span both at baseline and at follow-up at 48 weeks compared to those with mutations upstream of *DMD* exon 30 (Figure 3a), upstream of *DMD* exon 45 (Figure 3b), and upstream of *DMD* exon 63 (Figure 3c). The location of the nmDMD mutations by *DMD* exon are listed in Table 3. As shown, the number of subjects with nmDMD mutations upstream and downstream of *DMD* exon 30 were 26 and 29, respectively. When nmDMD mutations were assessed upstream and downstream of *DMD* exon 45, the number of subjects were 38 and 17, respectively. Mutations downstream of *DMD* exon 30 and *DMD* exon 45 will affect the expression of the shorter dystrophin isoforms dp260 and dp140, respectively. Nine subjects had nmDMD downstream of *DMD* exon 63 that affect the expression of dp71, and 46 subjects had nmDMD upstream of *DMD* exon 63.

Over the 48-week evaluation, the mean normalized forward and backward digit spans did not differ significantly based on age (*p* value 0.5) or on nmDMD mutation location (*DMD* exon 30 (*p* value 0.6), *DMD* exon 45 (*p* value 0.9), and *DMD* exon 63 (*p* value 0.5)).

**Figure 4:**
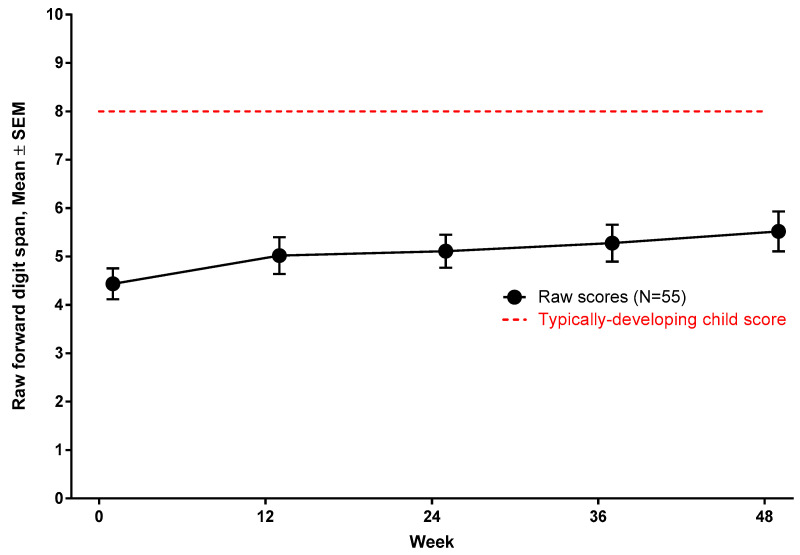
Developmental maturation of working memory in nmDMD.

To determine that developmental attenuation in working memory is not due to developmental regression or poor effort during testing, the raw scores of forward digit span were evaluated at baseline and at 48 weeks. Subjects made gains, albeit marginal, in forward digit span over 48 weeks (Figure 4).

### 3.3. Developmental Growth of Working Memory Based on nmDMD Mutation Location

**Figure 5:**
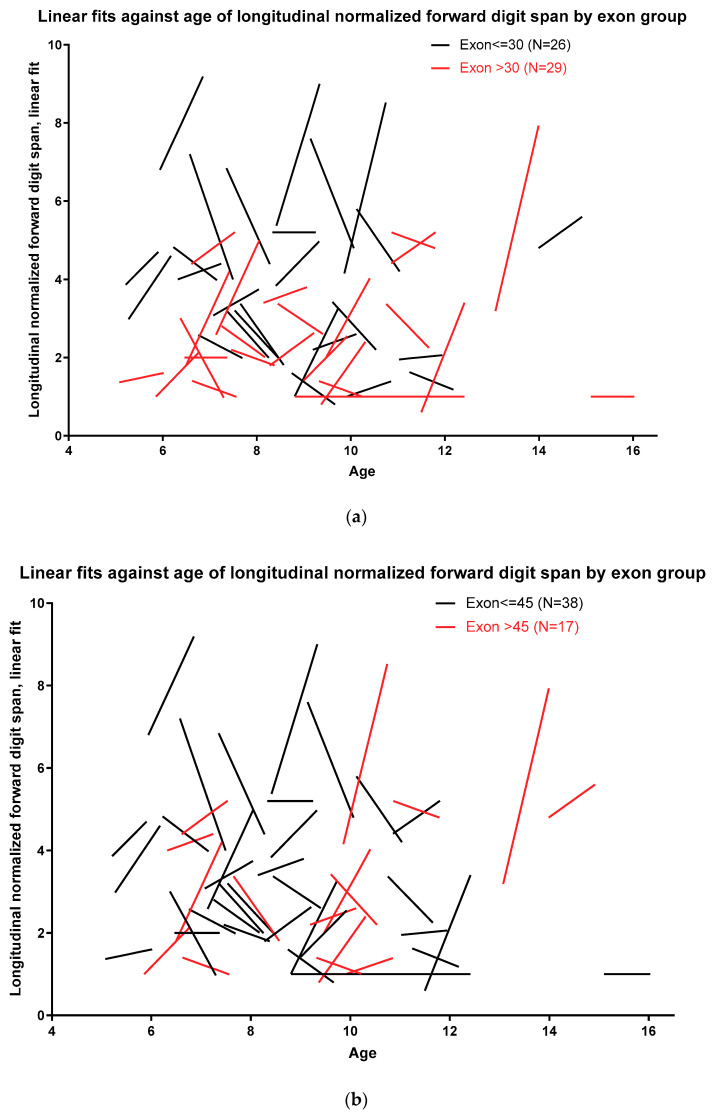

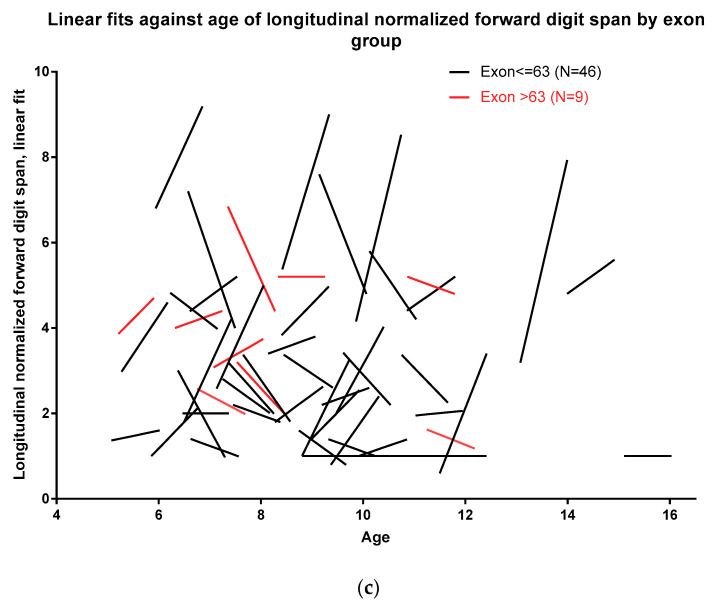
(**a**–**c**) Developmental modeling of forward digit span based on nonsense *DMD* (nmDMD) mutation location.

To model developmental growth, normalized forward digit spans at all five assessments were time-fitted using a linear growth model. This modeling was performed by categorizing nmDMD mutation location into nmDMD mutations upstream and downstream of *DMD* exon 30, *DMD* exon 45, and *DMD* exon 63. Figure 5a shows that, as a cohort, subjects with nmDMD mutation upstream of *DMD* exon 30 have better normalized forward digit span scores than subjects with mutations downstream of *DMD* exon 30. Similarly, subjects with nmDMD mutation upstream of *DMD* exon 45 and upstream of *DMD* exon 63 scored better on normalized forward digit span scores than subjects with mutations downstream of *DMD* exon 45 and downstream of *DMD* exon 63 (Figure 5b,c).

## 4. Discussion

We had shown earlier that subjects with nmDMD mutations downstream of *DMD* exon 45 perform poorly in forward and backward digit spans [34]. In this study, we extend our earlier cross-sectional findings and present scientific evidence that developmental gains in working memory in nmDMD are marginal and continue longitudinally but that developmental gains differ based on nmDMD mutation location. Subjects with nmDMD mutations upstream of *DMD* exon 45 demonstrate better gains in working memory compared to subjects with mutations downstream of *DMD* exon 45.

We found that, as a whole group, subjects with nmDMD mutations scored two standard deviations below the mean on forward digit span. Only 16 of the subjects in our cohort scored one standard deviation below the mean on forward digit span. This finding is striking. Leaffer et al. evaluated 170 boys with *DMD* mutations and noted that the mean Z-score was −0.34 (SD 1.1), with range −2.3 to +3.3 (comparable scaled score ranging from 4 to 16) signifying that performance ranges from being deficient to very superior [33]. One possible explanation as to why our study results are different from that of Leaffer et al. is that, in line with the reading-frame rule, nmDMD mutations tend to have severe disease phenotype [40,41]. Also, noteworthy is that Leaffer et al. did not distinguish between different *DMD* mutation locations, *DMD* mutation types, or age.

Our data show that subjects with nmDMD have significant deficits in working memory compared to typically developing peers, with older subjects (≥9 years) actually performing comparatively poorer than the younger subjects, though this difference did not reach statistical significance. Typically, developing children between ages 9–10 years can hold 5–6 digits span, reaching an adult capacity at an average of 7 digits span by ages 12–13 years [42]. Could low working memory score, as seen in our cohort, be simply reflective of global cognitive delay in nmDMD? If so, we would expect that the gap between actual performance and age-based accentuation increases with age due to cumulative impact of attenuated developmental progress. A developmental stagnation could plausibly explain why older subjects underperformed compared to the younger group on working memory score. While we cannot exclude with certainty that our cohort had lower intellectual capacity, as a gold-standard neuropsychological assessment was not performed concurrently, a seminal study by Hinton et al. showed that working memory is a cognitive domain that is disproportionately vulnerable in DMD, regardless of intellectual capacity [32].

Converging evidence from developmental cognitive neuroscience and imaging studies show that there is substantial improvement in working memory during infancy, childhood, and adolescence, reaching developmental maturity by late adolescence [43]. Visuospatial working memory maturation parallels progressive myelination of the frontoparietal areas that increases from late childhood through young adulthood. There are distinct neural substrates for working memory in children compared to adults. Ventrolateral prefrontal cortex is activated in both children and adults during working memory tasks, while adolescents and adults (ages 12 and above) recruit the dorsolateral prefrontal cortex [44]. Likewise, using functional MRI in a large cohort of children and young adults, ages 8–22 years, Kolskar demonstrated that, with developmental maturation, global effectiveness of connectivity improves with efficiency in transfer of information across global networks [45]. This maturation resembles network connectivity during tasks of executive function in adults. Thus, developmental maturation of cognition is associated with an increasing focused processing network [46]. Against this backdrop, our results suggest that the absence of dystrophin in the brain has negative impacts on the development of frontoparietal networks. While the clinical trial of our study did not include concurrent imaging, Doorenweerd et al. noted that boys with DMD and in particular those with mutations downstream of *DMD* exon 45 had smaller total and gray matter volume and lower white matter fractional anisotropy and performed worse on psychometric measures [25]. Similar to their study and congruent with our initial observations [34], we found that the longitudinal change in working memory was influenced by the location of the nmDMD mutation. Subjects with nmDMD mutations downstream of *DMD* exon 30, downstream of *DMD* exon 45, and downstream of *DMD* exon 63 showed lower scores in forward digit span both at baseline and at follow-up. Developmental growth modeling showed that subjects with nmDMD mutations upstream of *DMD* exon 30, upstream of *DMD* exon 45, and upstream of *DMD* exon 63 appeared to make better gains in working memory than subjects with mutations downstream of *DMD* exon 30, downstream of *DMD* exon 45, and downstream of *DMD* exon 63. Collectively, these data suggest specific roles for full-length dystrophin and shorter dystrophin proteins in brain development and brain network connectivity. Without overinterpreting our findings, we postulate that the cumulative loss of tissue-specific full-length and shorter dystrophin proteins may account for the lower digit span scores. For example, the absence of dystrophin in neurons and glial cells may alter neuronal-glial communication and may compromise neural connectivity. It can be hypothesized that *DMD* mutations affecting the expression of dystrophin dp140 can affect the neural circuity serving working memory.

We found that digit span stayed stable or continued to show slight improvement over time in all subjects. An important context while considering neuropsychological performance over time in children is the extent of practice effects. Earlier studies have shown that tests that assess semantic memory and word fluency show more practice effects compared to digit span [47,48]. Likewise, Lezak et al. noted that “tests that have a large speed component require an unfamiliar or infrequently practiced mode of response or have a single solution—particularly if it can be easily conceptualized once it is attained”—and are more likely to show practice effects [49]. Slade et al. evaluated approximately 500 children between ages 8–12 years to study the developmental maturation and practice effects of certain neuropsychological measures, including digit span [50]. These authors noted age-related increases in raw scores in digit span, with minimal susceptibility of digit span to practice effects, a finding reported by multiple research groups [48,51,52]. Thus, our data demonstrating stable or slight improvements in forward digit span over 48 weeks is unlikely to be due to practice effects. Hellebrekers et al. in a recent publication demonstrated a developmental stagnation in verbal span capacity in DMD, especially in boys with mutations downstream of *DMD* exon 45 [53].

Cognitive rehabilitation strategies have not been systematically evaluated in DMD. Our data suggests that individuals with DMD do make gains in working memory over time, although they differ in their developmental skills compared to typically developing children. This finding is encouraging and suggests that the window of intervention may be longer than previously thought in DMD. Among cognitive skills, working memory is “malleable” and can be improved by cognitive training. Several childhood medical conditions are associated with impairment in working memory including sickle cell disease, epilepsy, attention-deficit hyperactivity disorder, and childhood cancer survivors [35,54,55,56,57,58]. Intervention studies in these cohorts have improvements in working memory following cognitive training. The importance of working memory is that it is critical for learning and for academic success. Working memory capacity and function positively correlate to achievement tests in mathematics, reading, comprehension, speech, and computational skills [59,60]. Academic achievements are significantly lower in boys with DMD compared to their age-matched siblings (and accounting for environmental factors) [61]. Boys with DMD scored 10 points lower than the mean standard score in reading, writing, and mathematics, with mathematics showing the largest overall effect. There is further emerging evidence that executive function, the cognitive domain in which working memory is one of the principal components, determines health maintenance. Better executive functioning allows a mature ability to forecast future self or future consequences in light of immediate and salient competing or behavioral options [62]. Individual differences in working memory have been shown to relate to myopic versus disciplined health behaviors [63,64].

There are several strengths in our study. We conducted serial digit span measurement in a group of large number of subjects, all of whom are genetically homogenous. Additionally, the study evaluated subjects with DMD across a wide developmental epoch (ages 5 to 16 years). These attributes provide a “natural history of forward digit span” in nmDMD and will allow comparison to change following an intervention. There are also some limitations to our study. First, this clinical trial was not designed to evaluate cognition in a comprehensive manner, and therefore, our data is limited only to indices of verbal working memory. A full neuropsychological evaluation was not performed in the clinical trial participants. We acknowledge that comprehensive evaluation of several domains of cognition is pertinent in this population and should not be restricted only to indices of verbal working memory.

Our study is contemporaneous for several reasons. The average life expectancy in DMD now extends to the third-to-fourth decade of life [65,66], and corticosteroids and standard-of-care guidelines addressing the many facets of the disease have improved clinical outcomes in DMD. Adeno-associated-virus-based gene therapy to restore skeletal and cardiac health is currently in phase 2 clinical trial; this new treatment frontier combined with approval of newborn screening for DMD by the Food and Drug Administration Agency offers the opportunity to strategize treatment options for those showing cognitive deficits while planning for ways to prevent developmental problems before they arise [67]. The research intensity in DMD continues to be focused overwhelmingly on skeletal and cardiac health. Recognizing that cognition plays a role in health maintenance and affects long-term health outcomes urges us to reassess research priorities in this population. Lastly, cognitive clinical outcome measures are needed in DMD and will meet some of the unmet needs of this population.

While we continue to make tremendous progress, several fundamental questions remain: (i) what is the time course of developmental increase in cognitive ability in DMD; (2) how does the structure of cognition change with age; and (3) how do changes in different cognitive domains affect each other? Some potential suggestions included the following: First, consensus on the frequency of neuropsychological evaluation across the lifespan is needed. Young boys are more likely to be screened, given the developmental and cognitive concerns that they may present with. However, academic performance in this cohort is poor [61], suggesting that these boys may benefit from additional screening. Second, uniformity in the assessment of cognition is mandatory. Some of the neuropsychological measures used in earlier studies do not offer a continuity of measurement. In a lead study, we show that the NIH Toolbox—a scientifically robust neurobehavioral health measure—is a valid analytic tool for cognitive surveillance in DMD [68]. Last, well-powered natural history studies of cognition through a team-science approach will be extremely beneficial in DMD.

## Figures and Tables

**Table 1:** Summary of normalized forward digit span over time in subjects.

Time of Evaluation	Number of Subjects	Mean, Median (SD)	Range
Week 0	55	2.84, 3.00 (1.68)	1–7
Week 12	52	3.19, 2.50 (2.47)	1–12
Week 24	55	3.16, 3.00 (2.00)	1–8
Week 36	54	3.15, 2.50 (2.10)	1–10
Week 48	52	3.15, 3.00 (2.21)	1–10

**Table 2:** Summary of normalized backward digit span over time in subjects.

Time of Evaluation	Number of Subjects	Mean, Median (SD)	Range
Week 0	54	1.17, 1.00 (0.54)	1–4
Week 12	52	1.23, 1.00 (0.73)	1–5
Week 24	54	1.22, 1.00 (0.77)	1–5
Week 36	53	1.13, 1.00 (0.52)	1–4
Week 48	52	1.19, 1.00 (0.66)	1–4

**Table 3:** The location of nmDMD mutation by the *DMD* exon is listed.

Location of nmDMD Mutation by *DMD* Exon
3
4
6
7
7
7
11
12
12
14
14
15
16
18
19
20
21
21
22
23
23
24
24
25
29
29
33
33
33
35
35
38
39
40
40
44
44
44
52
55
55
55
59
60
61
61
65
66
66
68
68
70
70
70
70
70
70

## Supplementary Materials

The following are available online at https://www.mdpi.com/2077-0383/9/9/2940/s1, Supplementary Table S1. Co-investigators from Study PTC124-GD-007-DMD, Supplementary Table S2. The Clinical Evaluator Training Group for Study PTC124-GD-007-DMD.
