# Supplementary material for: Longitudinal Evaluation of Working Memory in Duchenne Muscular Dystrophy
Source: J Clin Med. 2020 Sep 11;9(9):2940. doi: 10.3390/jcm9092940 (PMC7563441; doi:10.3390/jcm9092940)
Supplement: Supplementary file 1 [file jcm-09-02940-s001.pdf]

SUPPLEMENTARY MATERIAL

Supplementary Table S1. Co-investigators from Study PTC124-GD-007-DMD.

Name	Location	Role	Contribution
Susan D. Apkon, MD	Children's Hospital Colorado, Aurora, Colorado, USA	Site Investigator	Enrolled patients in Study PTC124-GD-007-DMD
Richard J. Barohn, MD	University of Kansas Medical Center, Kansas City, Kansas, USA	Site Investigator	Enrolled patients in Study PTC124-GD-007-DMD
Enrico Bertini, MD	Medicina Moleculare di Malattie Neuromuscolari e Neurodegenerative Dipartimento dei Laboratori Ospedale Pediatrico Bambino Gesù di Roma, Rome, Italy	Site Investigator	Enrolled patients in Study PTC124-GD-007-DMD
Stephanie Burns-Wechsler, MD	Duke Clinical Research Unit Rankin Ward, Durham, North Carolina, USA	Site Investigator	Enrolled patients in Study PTC124-GD-007-DMD
Katherine M.D. Bushby, MD	Clinical Research Centre 4th Floor Leazes Wing, Newcastle-upon-Tyne, UK	Site Investigator	Enrolled patients in Study PTC124-GD-007-DMD
Craig Campbell, MD	Department of Pediatrics, Children's Hospital Ontario, University of Western Ontario, London, Ontario, Canada	Site Investigator	Enrolled patients in Study PTC124-GD-007-DMD
Brigitte Chabrol, MD	Neurologie Pediatrique, Unité de Médecine Infantile Hôpital d'Enfants, Marseille, France	Site Investigator	Enrolled patients in Study PTC124-GD-007-DMD
Emma Ciafaloni, MD	University of Rochester Medical Center, Department of Neurology, Rochester, New York, USA	Site Investigator	Enrolled patients in Study PTC124-GD-007-DMD
Jaume Colomer, PhD	Departamento de Neuropediatria, Hospital Sant Joan de Deu, Barcelona, Spain	Site Investigator	Enrolled patients in Study PTC124-GD-007-DMD
Giacomo Pietro Comi, MD	Unita Operativa di Neurologia Fondazinone IRCCS, Ospedale Maggiore Policlinico, Milan, Italy	Site Investigator	Enrolled patients in Study PTC124-GD-007-DMD
Anne Connolly, MD	Washington University, St. Louis, Missouri, USA	Site Investigator	Enrolled patients in Study PTC124-GD-007-DMD
Basil T. Darras, MD	Boston Children's Hospital, Harvard Medical School, Boston, Massachusetts, USA	Site Investigator	Enrolled patients in Study PTC124-GD-007-DMD
John W. Day, MD	University of Minnesota Clinical and Translational Science Institute, Minneapolis, Minnesota, USA	Site Investigator	Enrolled patients in Study PTC124-GD-007-DMD
Richard S. Finkel, MD	Children's Hospital of Philadelphia, Philadelphia, Pennsylvania, USA	Site Investigator	Enrolled patients in Study PTC124-GD-007-DMD
Kevin M. Flanigan, MD	University of Utah Medical Center, Salt Lake City, Utah, USA	Site Investigator	Enrolled patients in Study PTC124-GD-007-DMD
Nathalie Goemans, MD	University Hospital Leuven, KU Leuven, Leuven, Belgium	Site Investigator	Enrolled patients in Study PTC124-GD-007-DMD

Susan T. Iannaccone, MD	University of Texas Southwestern Medical Center, Children's Medical Center, Dallas, Texas, USA	Site Investigator	Enrolled patients in Study PTC124-GD-007-DMD
Kristi J. Jones, MD	Kids Neuroscience, The Children's Hospital at Westmead, Westmead, New South Wales, Australia	Site Investigator	Enrolled patients in Study PTC124-GD-007-DMD
Petra Kaufmann, MD	Columbia University Pediatric Neuromuscular Center, New York, New York, USA	Site Investigator	Enrolled patients in Study PTC124-GD-007-DMD
Janbernd Kirschner, MD	Department of Neuropediatrics and Muscle Disorders, Medical Center – University of Freiburg, Freiburg, Germany	Site Investigator	Enrolled patients in Study PTC124-GD-007-DMD
Jean K. Mah, MD	Alberta Children's Hospital, NW Calgary, Alberta, Alberta, Canada	Site Investigator	Enrolled patients in Study PTC124-GD-007-DMD
Katherine D. Mathews, MD	Institute for Clinical and Translational Science, General Hospital, Iowa City, Iowa, USA	Site Investigator	Enrolled patients in Study PTC124-GD-007-DMD
Craig M. McDonald, MD	Department of Physical Medicine & Rehabilitation, The University of California, Davis Medical Center, Sacramento, California, USA	Site Investigator	Enrolled patients in Study PTC124-GD-007-DMD
Eugenio Mercuri, MD	Dipartimento di Sciencze Pediatriche Medico-Chiurgiche e Neuroscienze dello Svilippo U.O. Complessa di Neurospichiatria Infantile Policlinica Universitario A. Gemelli, Rome, Italy	Site Investigator	Enrolled patients in Study PTC124-GD-007-DMD
Francesco Muntoni, MD	University College London Great Ormond Street Institute of Child Health, London, UK	Site Investigator	Enrolled patients in Study PTC124-GD-007-DMD
Yoram Nevo, MD	Schneider Children's Medical Center, Tel Aviv University, Tel Aviv, Israel	Site Investigator	Enrolled patients in Study PTC124-GD-007-DMD
Julie Parsons, MD	Children's Hospital Colorado, Aurora, Colorado, USA	Site Investigator	Enrolled patients in Study PTC124-GD-007-DMD
Yann Péréon, MD, PhD	Reference Centre for Neuromuscular Disorders AOC, Hôtel-Dieu, Nantes, France	Site Investigator	Enrolled patients in Study PTC124-GD-007-DMD
Rosaline C.M. Quinlivan, MD	Robert Jones & Agnes Hunt Orthopaedic Hospital NHS Trust, Children's Unit, Gobowen Oswestry, UK	Site Investigator	Enrolled patients in Study PTC124-GD-007-DMD
J. Ben Renfroe, MD	Northwest Florida Clinical Research Group, Gulf Breeze, Florida, USA	Site Investigator	Enrolled patients in Study PTC124-GD-007-DMD
Barry S. Russman, MD	Shriners Hospitals for Children, Portland, Oregon, USA	Site Investigator	Enrolled patients in Study PTC124-GD-007-DMD
Monique M. Ryan, MD	The Royal Children's Hospital, Parkville, Victoria, Australia	Site Investigator	Enrolled patients in Study PTC124-GD-007-DMD
Jacinda B. Sampson, MD PhD	University of Utah Medical Center, Salt Lake City, Utah, USA	Site Investigator	Enrolled patients in Study PTC124-GD-007-DMD
Ulrike Schara, MD	Department of Pediatric Neurology, University of Essen, Essen, Germany	Site Investigator	Enrolled patients in Study PTC124-GD-007-DMD

Thomas Sejersen, MD	Department of Neuropediatrics, Karolinska University Hospital, Stockholm, Sweden	Site Investigator	Enrolled patients in Study PTC124-GD-007-DMD
Kathryn Selby, MD	Children’s & Women’s Health Centre of British Columbia & University of British Columbia, Vancouver, British Columbia, Canada	Site Investigator	Enrolled patients in Study PTC124-GD-007-DMD
Douglas M. Sproule, MD	Columbia University Pediatric Neuromuscular Center, New York, New York, USA	Site Investigator	Enrolled patients in Study PTC124-GD-007-DMD
Már Tulinius, MD	Department of Pediatrics, Queen Silvia Children’s Hospital, University of Gothenburg, Gothenburg, Sweden	Site Investigator	Enrolled patients in Study PTC124-GD-007-DMD
Thomas Voit, MD	Institiut de Myologie, Groupe Hospitalier Pitie-Salpetriere, Paris, France	Site Investigator	Enrolled patients in Study PTC124-GD-007-DMD
Juan J. Vílchez, MD	Departamento de Neurologia, Hospital Universitario La Fe, Valencia, Spain	Site Investigator	Enrolled patients in Study PTC124-GD-007-DMD
Brenda L. Wong, MD	Cincinnati Children's Hospital Medical Center, Cincinnati, Ohio, USA	Site Investigator	Enrolled patients in Study PTC124-GD-007-DMD

Supplementary Table S2 The Clinical Evaluator Training Group for Study PTC124-GD-007-DMD.

Name	Location	Role	Contribution
R. Ted Abresch, MS	University of California Davis, School of Medicine, Sacramento, California, USA	Clinical Evaluator Training Group (PTC124-GD-007-DMD)	Responsible for the development of standardized procedures for the 6MWT, TFTs, myometry, and StepWatch® Activity Monitor calibration; development of the Clinical Evaluator manual; and overseeing training of site Clinical Evaluators.
Kim Coleman, MS	OrthoCare Innovations, Mountlake Terrace, Washington, USA	Clinical Evaluator Training Group (PTC124-GD-007-DMD)	Responsible for the development of standardized procedures for the 6MWT, TFTs, myometry, and StepWatch® Activity Monitor calibration; development of the Clinical Evaluator manual; and overseeing training of site Clinical Evaluators.
Michelle Eagle, PhD, MSc, MCSP	Atom International, Gateshead, UK	Clinical Evaluator Training Group (PTC124-GD-007-DMD)	Responsible for the development of standardized procedures for the 6MWT, TFTs, myometry, and StepWatch® Activity Monitor calibration; development of the Clinical Evaluator manual; and overseeing training of site Clinical Evaluators.
Eduard Gappmaier, PT, PhD	University of Utah Hospital, General Clinical Research Center, Salt Lake City, Utah, USA	Clinical Evaluator Training Group (PTC124-GD-007-DMD)	Responsible for the development of standardized procedures for the 6MWT, TFTs, myometry, and StepWatch® Activity Monitor calibration; development of the Clinical Evaluator manual; and overseeing training of site Clinical Evaluators.
Allan M. Glanzman, PT, DPT, PCS, ATP	The Children's Hospital of Philadelphia, Philadelphia, Pennsylvania, USA	Clinical Evaluator Training Group (PTC124-GD-007-DMD)	Responsible for the development of standardized procedures for the 6MWT, TFTs, myometry, and StepWatch® Activity Monitor calibration; development of the Clinical Evaluator manual; and overseeing training of site Clinical Evaluators.
Julaine Florence, PT, DPT	Washington University School of Medicine, St. Louis, Missouri, USA	Clinical Evaluator Training Group (PTC124-GD-007-DMD)	Responsible for the development of standardized procedures for the 6MWT, TFTs, myometry, and StepWatch® Activity Monitor calibration; development of the Clinical Evaluator manual; and overseeing training of site Clinical Evaluators.
Erik Henricson, MPH	University of California Davis, School of Medicine, Sacramento, California, USA	Clinical Evaluator Training Group (PTC124-GD-007-DMD)	Responsible for the development of standardized procedures for the 6MWT, TFTs, myometry, and StepWatch® Activity Monitor calibration; development of the Clinical Evaluator manual; and overseeing training of site Clinical Evaluators.

6MWT, 6-minute walk test; NSAA, North Star Ambulatory Assessment; TFT, timed-function.
